# Illusory Oscillation of the Central Rotation Axis

**DOI:** 10.1177/2041669519865283

**Published:** 2019-07-25

**Authors:** Yutaka Nakajima, Shohei Kakuda, Shunji Satoh

**Affiliations:** Seikei University, Tokyo, Japan; The University of Electro-Communications, Tokyo, Japan; The University of Electro-Communications, Tokyo, Japan

**Keywords:** visual illusion, rotational motion, oscillation, modal/amodal completion

## Abstract

In this study, we report a novel visual illusion for rotational motion, in which the central rotation axis of a partially invisible (apparent) square is perceived as exhibiting oscillatory rotation. To investigate the cause of this illusion, we measured the central position of a static apparent shape using an adjustment method (Experiment 1) and manipulated the speed of the rotating apparent square to test whether the illusion could be cancelled out by counteracting rotation using a constant method (Experiment 2). The results revealed that the perceived central position of a static apparent shape was shifted toward the outside. The shifted position depended on the orientation of the stimulus, and its position was arranged as if it was moving in a circular trajectory. In addition, the cancellation technique using counteracting rotation was successful, and cancellation of faster rotation required a greater radius of counteracting rotation. These results indicated that the illusion is induced by an interaction between illusory shifts of the central position of the static shape and the summation of motion vectors or motion momentum (e.g., centrifugal force) derived from shape representation by perceptual completion.

## Introduction

**Figure 1.**
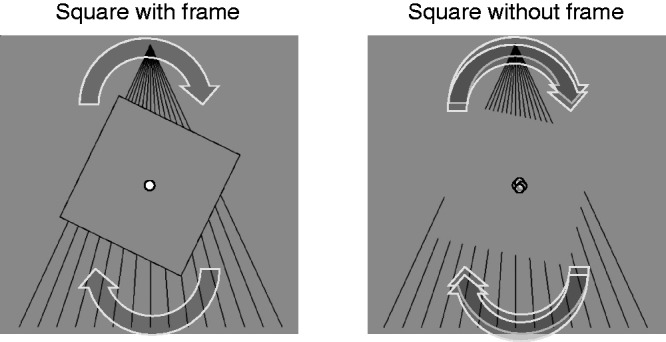
Schematic diagram of illusory oscillation of the central rotation axis (OCRA). When the square with a frame rotates with a fixed central rotation axis, observers perceive rotation of the square (left panel). In this situation, if the contour of the square diminishes, the perception of the square without a frame (apparent square) can be maintained. However, observers often also perceive illusory oscillation of the central rotation axis of the square (right panel). In the actual stimuli, the small white circle is not shown.

We report a novel visual illusion involving the illusory perception of a moving shape (Figure 1 and Movie 1). When a square with a frame rotates on a fan-shaped background pattern with a centre (i.e., the intersection of two diagonals) that is consistent with the central rotation axis, the square can be perceived as rotating at a fixed position. When the frame disappears, some parts (vertices and contours) of the square are still visible on the background pattern, whereas other parts outside the background pattern become invisible because the luminance of the square and background are the same. Preliminary observations in our laboratory have revealed that the perception of this rotating square can remain constant. Moreover, when the rotating square is perceived, the central rotation axis is also perceived, as if the axis is oscillating. The oscillation appears to have some regularity. Specifically, when the square was rotated without the frame, some observers reported that the oscillation was perceived as rotating in the same direction as that of the rotating square, not as random oscillation of the axis (e.g., if the square rotated in a clockwise direction, the perceived direction of the central rotation axis was also clockwise). Observing the video clip shown in Movie 1 can generate this perception. We call this phenomenon illusory oscillation of the central rotation axis (OCRA).

**Movie 1.** Movie of illusory oscillation of the central rotation axis (OCRA).*Note*: Details are described in the main text and in the caption of Figure 1.

One of the important features required for the perception of OCRA is the occurrence of perceptual completion. Some previous studies have reported an integrated relationship between perceptual shape completion and motion perception. It is well known that a rotating framed square can be perceived, even when all vertices are occluded (motion binding with some contours; Lorenceau & Shiffrar, 1992). For such stimuli, the motion vectors of each contour between occluding surfaces would be expected to be ambiguous, and the phenomena would therefore appear to be related to the aperture problem (Shimojo, Silverman, & Nakayama, 1989). In addition, such ambiguous motion vectors would be expected to be integrated with various constraints (e.g., shape information; Lorenceau & Alais, 2001; Lorenceau & Lalanne, 2008).

Compared with these previous phenomena, OCRA might represent a slightly different type of illusion, in which the upper invisible part of the square is modally completed, induced by the lower part of the square (Anderson, Singh, & Fleming, 2002). Although this is not really a case of occlusion, in the case of OCRA, upper invisible portions may be interpolated from the visible portions (i.e., a modally completed contour at the lower portion). In this sense, the upper part would be completed modally as vertices, suggesting a similarity between OCRA and motion binding stimuli. However, general motion binding stimuli cannot induce the perception of oscillating rotation. In addition, OCRA would presumably be less closely related to the aperture problem of motion vectors. Specifically, motion of lower vertices can be visible. The motion vectors for these vertices can be determined in one direction.

Another type of motion illusion related to perceptual completion is the breathing illusion (Bruno, 2001; Meyer & Dougherty, 1990; Shiffrar & Pavel, 1991), which is perceived when a rotating square consists of a stimulus configuration similar to a Kanizsa square. Thus, a rotating square with the same luminance as the background is perceived as alternately expanding and contracting. Thus, the illusion can be induced by alternation of occlusion and nonocclusion states for the vertices of the square. This induction method has similar elements to that of OCRA. In the stimuli used to induce the breathing illusion, all vertices are occluded (or not occluded) at the same time. In contrast, the induction stimuli in OCRA are not; there is a different timing of the disappearance of each vertex due to a lack of contrast against the background. In addition, as in the breathing illusion, changes in the speed of rotation can also be perceived with the perception of expansion and contraction of the square. However, OCRA stimuli do not appear to induce this type of perception, because the changing speed of the stimuli is not perceived in the illusion. This suggests that OCRA cannot be explained by the aperture problem for motion vector that explains the occurrence of the breathing illusion (Shiffrar & Pavel, 1991). Rather, OCRA appears to be induced by an additional information source: static cues and interpolation of the shape itself.

The current study was conducted to investigate the mechanisms underlying OCRA by quantitatively measuring the amount of perceived OCRA during the illusion. In Experiment 1, we measured the subjective centre in the static square without a frame (apparent square) using the adjustment method to investigate whether the stimulus configuration itself would have an effect on such oscillatory perception. In Experiment 2, we adopted a constant method (i.e., a cancellation technique). We presented various types of counteracting rotation during OCRA to investigate what kind of rotation could cancel this illusion. Thus, we investigated the effects of static and motion information on OCRA independently.

## Experiment 1

### Methods

#### Participants

Seven volunteers (two females and five males) from the University of Electro-Communications participated in the experiment (age [mean ± *SD*]: 25.0 ± 4.3 years). All participants had normal or corrected-to-normal vision and none had any neurological or visual disorders. Participants provided written informed consent, and all experimental protocols were reviewed and approved by the ethical committee of the University of Electro-Communications. All experiments were performed in accordance with the Code of Ethics of the World Medical Association (Declaration of Helsinki).

#### Apparatus

Stimuli were generated using Psychlops software (http://psychlops.osdn.jp, see also Maruya et al., 2010) on a Precision T5610 Workstation (Dell, Inc., Round Rock, TX) with Quadro K2000 (NVIDIA Corp., Santa Clara, CA). Stimuli were displayed on a 21-in. CRT monitor (GDM-F520; SONY Corp., Japan; spatial resolution: 1920 pixels × 1440 pixels, 48 cm × 31 cm, refresh rate: 80 Hz). The monitor was gamma-corrected to obtain a linear luminance output. All experiments were conducted in a dark room lit only by the monitor, at a viewing distance of 80 cm, which was kept constant using a chin rest.

#### Stimuli

**Figure 2.**
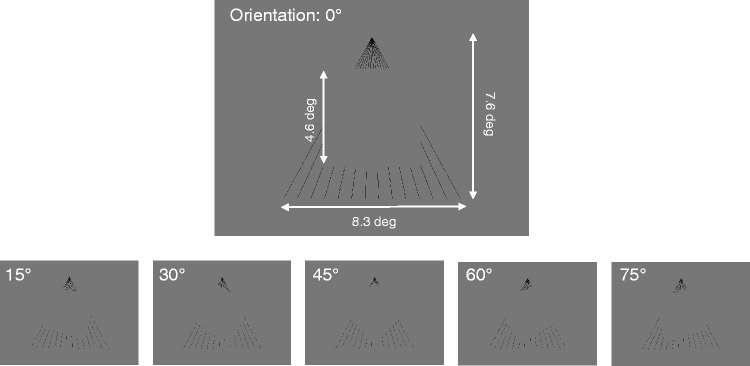
Stimuli in Experiment 1. A square without a frame (apparent square) was presented on a fan-like background pattern consisting of 14 lines. In the experiment, we manipulated the orientation of the square, ranging from 0° to 82.5°, at an interval of 7.5° steps. The figure shows six examples of the orientation conditions.

The stimuli consisted of a background pattern and a square without a frame (apparent square). The background pattern was shaped like a fan, similar to a half segment of the induction stimuli used in the Hering illusion (Figure 2). The 14 black lines (0.03 cd/m^2^) radiated from a point located above the square. The height and width of the pattern were 7.6 deg and 8.3 deg, respectively. An apparent square was presented at the centre of the pattern. The luminance of the square was equal to that of the background (i.e., mean luminance: 36.8 cd/m^2^), meaning that some vertices and contours of the square were invisible, depending on its orientation. The square was 4.6 deg × 4.6 deg in size and was drawn with the luminance of the background (36.8 cd/m^2^) using Psychlops software (Maruya et al., 2010). The orientation of the square was randomly assigned from 0° to 82.5°at an interval of 7.5°. The centre of the apparent square was consistent with that of the background pattern (Figure 2).

#### Procedure

In each trial, one of the oriented apparent squares was randomly presented. The position of the stimuli (consisting of an apparent square on the background pattern) was randomly located within a 1 deg^2^ region around the centre of the monitor. At that time, a small white square (a marker, 73.6 cd/m^2^) was also presented at a random position within 0.6 deg^2^ region around the centre of the monitor. Participants were asked to move the marker to the subjective centre of the square using a key press, until they found a position they felt was satisfactory. During each of 12 trial blocks, 12 conditions of orientation were presented once in a random order, for a total of 120 trials (10 trials × 12 blocks). As a control condition, we also measured the subjective centre of the square with a frame using the same procedure described earlier. The data from this condition can be regarded as reflecting the internal bias for each participant when judging the centre of the square in our stimulus configuration.

### Results and Discussion

**Figure 3.**
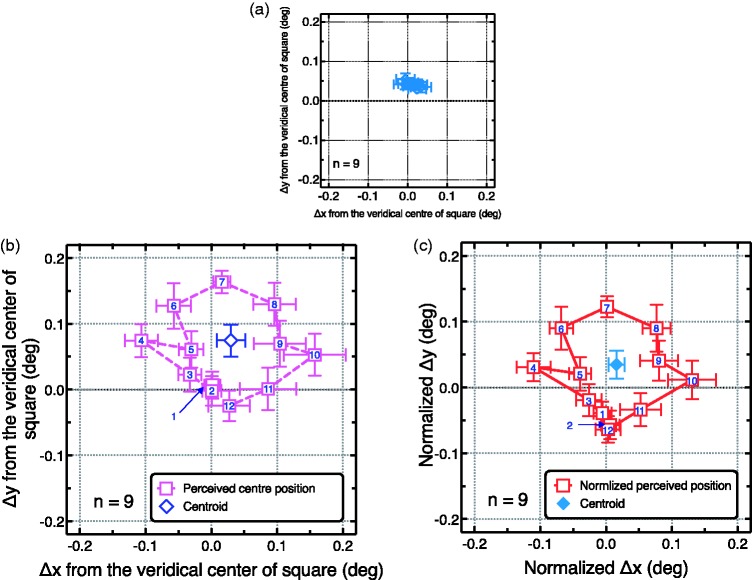
The results of Experiment 1. (a and b) Average differences between the subjective and veridical centre positions of the static square with and without a frame. The horizontal and vertical axes indicated the difference from the veridical centre of each square (Δx and Δy), respectively. The numbers in (b) (and (c)) at each data point indicate the orientation condition (1: 0°, 2: 7.5°, 3: 15°, 4: 22.5°, 5: 30°, 6: 37.5°, 7: 45°, 8: 52.5°, 9: 60°, 10: 67.5°, 11:74.5°, 12: 82.5°). We also calculated the centre of figure (centroid) from data points (◇)．We then normalized the results in (b) by subtracting the values from those in (a) (and (c)). Error bars in each panel indicate the standard error of the mean.

The data were represented in x–y coordinates. We calculated the difference between each x(y) position of the data and the veridical centre of the presented square for each participant. The average position of the subjective centre is shown in Figure 3(a) (square with frame) and 3(b) (square without frame). The results suggest that the subjective centre of the square with frame (Figure 3(a)) was consistently shifted upwards, while the horizontal offset varied between conditions. In contrast, the subjective centre of the square without a frame (Figure 3(b)) was plotted on an oval-like orbit (a circular path), indicating that the judgment of the subjective centre depended on the orientation of the presented square. To show the amount of the shift for the centre position and the length of the radius of the orbit, the centre of the orbit (i.e., centroid) and the mean radius (mean of the lengths between centroid and each data point) was calculated for each participant and averaged between participants (Figure 3(b)). One-sample t-tests for the centroid revealed that vertical position of centroid was significantly different from the origin—x of centre: *t*(8) = 0.86, *p *=* *.* *21; y of centre: *t*(8) = 2.62, *p* = .02 The radius was also significantly different from zero—*t*(8) = 7.50, *p* < .001. To eliminate the internal bias for judging the centre of the square shape, we normalized the results of the square without frame by subtracting the values from those of the square with frame, for each participant. The averaged results (Figure 3(c)) revealed that the general tendency did not differ, and each data point was slightly shifted downwards. Note that this normalized data did not indicate the perception of participants itself; rather, the perceived centre positions were shown in the unnormalised data (Figure 3(b)).

These results indicate that the subjective centre positions of a shape without a frame would be shifted. The amount (vector) of the shift corresponded to the orientation of the apparent square, as if the centre position itself rotated in an oval orbit. This finding suggests that the perceptual oscillation of OCRA exhibits a rotational pattern, and that a partially invisible square itself, even if static, contributes to the perception of OCRA. Another interpretation for the shift of the subjective centre position is that the perceptual completion of the apparent shape would not result in a square, but in a rectangle. This issue will be discussed in more depth in the General discussion section later.

## Experiment 2

The results of Experiment 1 suggested that the stimulus configuration itself (i.e., static information) contributes to OCRA. Specifically, we found that the centre of the apparent square was shifted, following a rotational pattern. This finding suggests that such a configuration induces an illusory shift of the centre position. If OCRA follows a rotational pattern with movement in an oval orbit as shown in Figure 3, we would expect it to be cancelled out by the addition of counteracting motion. To test this assumption, we adopted the cancellation technique in Experiment 2. Thus, we rotated the central rotation axis, and manipulated the length of horizontal and vertical radii of the rotation independently. If the perception of OCRA simply follows the perception of the subjective centre of the square, this manipulation would be expected to successfully cancel out the illusion, and the obtained radii resulting in the size of the circular orbit would be consistent with the results of Experiment 1.

### Methods

#### Participants

Six volunteers (all males) from the University of Electro-Communications, including the first author, participated in the experiment (age [mean ± *SD*]: 26.2 ± 4.9 years). All participants had normal or corrected-to-normal vision and none had any neurological or visual disorders. Participants provided written informed consent, and all experimental protocols were reviewed and approved by the ethical committee of the University of Electro-Communications. All experiments were performed in accordance with the Code of Ethics of the World Medical Association (Declaration of Helsinki).

#### Apparatus and Stimuli

**Figure 4.**
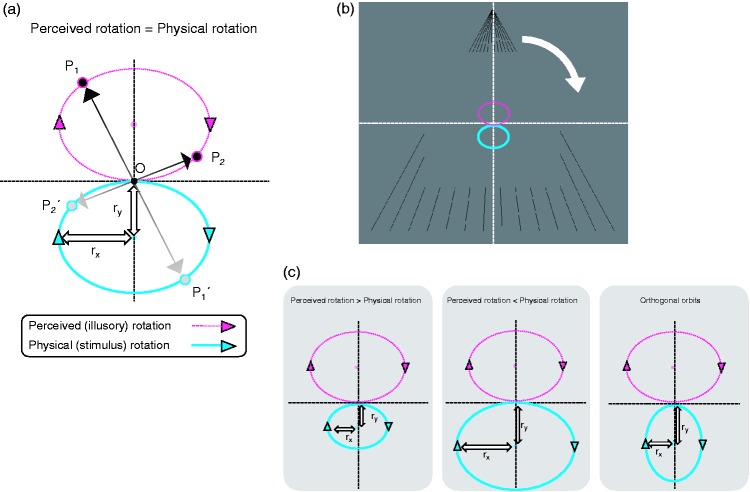
The manipulation of the radii (*r*_x_ and *r*_y_) of additional rotational components in Experiment 2. (a) A schematic diagram of the cancellation of rotational motion (upper pink dotted orbit) by adding another rotational motion (lower blue solid orbit). P1 and P2 indicate the arbitrary motion components, moving in a clockwise direction on a circular orbit (pink dotted orbit). P1 and P2 could generally be cancelled out by motion in point symmetry by O (intersection of two black dashed lines): P1´ and P2´, respectively. Therefore, P1´ and P2´ also moved in a clockwise direction on another circular orbit (blue solid orbit). Here, we regarded the pink circle as the orbit of the illusory-rotating central rotation axis in OCRA and the blue circle as the orbit of the physically rotating central axis of the stimuli ((b) and (c)). The curved white arrow in (b) indicates the rotational direction of the apparent square. By adding another motion (i.e., the motion shown by the blue orbit) and manipulating the radii of the orbit (*r*_x_ and *r*_y_), we assumed that OCRA could be cancelled out by radii of an appropriate length (b). In contrast, (c) shows that the added motion could not cancel the illusion due to inadequate radii.

The stimuli were the same as those in Experiment 1, except for the following differences: In Experiment 2, the square without a frame rotated at the speed of 1/4, 1/2, or 1 revolutions per second (rps). We defined each rotation as a baseline. The results of Experiment 1 suggest that the central rotation axis could be perceived as rotating in an oval orbit with a centre that could be shifted upwards from the veridical centre of the stimulus (Figures 1 and 4(b)). We next rotated the central rotation axis, such that the centre of the rotational orbit was shifted downwards, but the direction of the rotation was the same as the baseline rotation, corresponding to the motion in point symmetry with the illusory OCRA derived from each baseline motion (Figure 4). Therefore, the radius of rotation (*r*_x_ and *r*_y_) was manipulated independently. The length of *r*_x_ was 0.00, 0.09, 0.17, 0.26, 0.35, or 0.43 deg, and that of *r*_y_ was 0.00, 0.09, 0.17, 0.26, or 0.35 deg. Note that the y position of the centre of the orbit of additional rotation was changed depending on the *r*_y_ condition (Figure 4).

#### Procedure

In each trial, one of the rotating squares without a frame (apparent square) with randomly selected x and y radii was presented for 1 s. The position of stimuli was randomly positioned within a 1 deg^2^ region at the centre of the screen, as in Experiment 1. Participants were asked to judge whether the central rotation axis of the presented stimulus oscillated or not, using a constant method. In each block, the rotational speed of the square was fixed, and one of 30 combinations of the x and y radii was presented 10 times. The total number of trials was 600 (30 conditions of radii × 2 rotational directions × 10 repetitions) including a rest interval (per 100 trials).

### Results and Discussion

**Figure 5.**
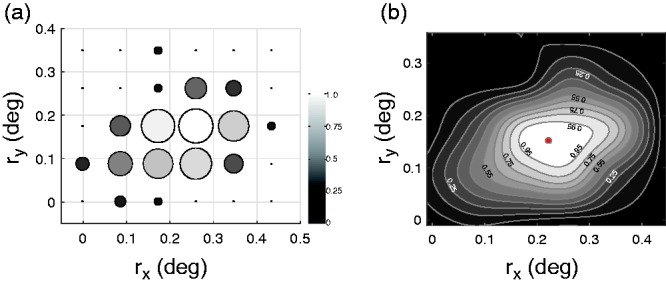
An example of the data in Experiment 2. These data were obtained from participant #2 in the ½ revolutions/s condition. The size of the circle in (a) shows the proportion of trials in which a participant perceived the presented stimuli as having a non-oscillating central rotation axis (i.e., the stability ratio for rotation; SRR). A brighter and larger circle indicates higher SRR values. Horizontal and vertical axes indicate the length of *r*_x_ and *r*_y_, respectively. (b) A contour plot of estimated SRR values derived from bicubic interpolation of the SRR results. The red circle in the white region indicates the estimated highest SRR for a participant.

**Figure 6.**
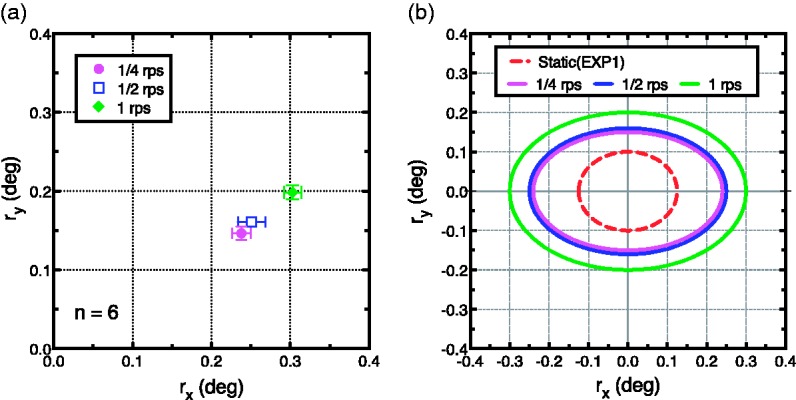
The average radii inducing the highest SRR for three speed conditions (a). The highest obtained SRRs for each speed condition (cf. Figure 5(b)) were averaged across six participants. The horizontal (vertical) axis indicates *r*_x_ (*r*_y_). Error bars indicate the standard deviation of the mean. (b) Estimated oval orbit of the illusory oscillation. The red dashed line indicates the estimated orbit from the results of Experiment 1 (Figure 1(c)). To clearly show the differences between conditions, the centre positions of the orbits are located at the origin.

We calculated the proportion of trials in which the stimuli were perceived as having a nonoscillating central rotation axis (stability ratio for rotation [SRR]) in each condition, for each participant. Figure 5(a) shows an example of the SRR values in the 1/2 rps condition. To estimate the radii that induced the highest SRR, we converted SRR into a 2D image by regarding SRR as a luminance value. Here, we adopted a bicubic interpolation method and estimated the highest SRR between the radii conditions for each participant. Figure 5(b) shows an example of the 2D contour plots of interpolated SRR values, with the highest SRR for the 1/2 rps condition. This procedure was conducted for each speed condition for each participant. In addition, we calculated the average highest SRR for each speed condition (Figure 6(a)).

Importantly, these results revealed that OCRA could be cancelled out by counteracting rotation. In addition, Figure 6(a) clearly shows that OCRA was speed-dependent, such that a faster speed of rotation required a larger radius of counter-rotation. In addition, perceived oscillation of OCRA followed an ellipsoidal orbit, such that the radius required to cancel OCRA was greater in the horizontal direction than in the vertical direction. The highest SRR values for 1/4, 1/2, and 1 rps conditions were obtained at (average *r*_x_, average *r*_y_) = (0.24, 0.15), (0.25, 0.16), and (0.30, 0.20), respectively. One-way analysis of variance shows the significant main effects of rotational speed for both *r*_x_ and *r*_y_—*r*_x_: *F*(2,17) = 8.93, *p* = .0022; *r*_y_: *F*(2,17) = 14.31, *p* = .0002. Multiple comparisons with Bonferroni correction for *r*_x_ and *r*_y_ revealed that each radius in 1 rps condition was significantly larger than those in the 1/2 and 1/4 conditions (*p* < .05, Bonferroni corrected).

Note that the range of subjective central positions measured in Experiment 1 was approximately 0.25 deg in the horizontal direction and approximately 0.20 deg in the vertical direction (cf. Figure 3). If these ranges are considered as the diameter of the orbit of rotation of the central rotation axis, the radius would be expected to be 0.125 deg (*r*_x_) and 0.1 deg (*r*_y_). These radii were smaller (approximately half the size) than those in even the slowest (1/4 rps) condition (Figure 6(b)). This finding suggests that rotational motion enhanced OCRA. The subjective oscillatory movement perceived in OCRA thus appears to consist of a shift of the centre position of the static shape and a continuous shift of the central rotation axis by rotational motion.

In summary, the results of Experiment 2 clearly demonstrated the speed dependency of OCRA, suggesting the contribution of motion information to the perception of OCRA.

## General Discussion

### Effect of Invisible Vertices on OCRA

The results of Experiment 1 suggested that the centre position of a square without a frame (apparent square) was perceived as shifted (Figure 3), even when the square was static. This finding suggests that static information contributes to the occurrence of OCRA. The perceptual positional shift of the centre of the static shape could be explained by perceptual distortion (expansion and contraction) due to the completion of the partially invisible shape (Palmer, Brooks, & Lai, 2007; Vezzani, 1999). Regarding the stimulus configuration of OCRA, when the vertices were invisible, the interpolated shape could be distorted from the square, causing the centre position of the square to be perceived as shifted. In the previous observation, the central rotation axis of the square without a frame could be perceived as rotating. The results of Experiment 1 appear to be consistent with this observation, revealing that the perceived centre positions for the oriented squares (Figure 3) were arranged in an oval-like path. These characteristics may also indicate the contribution of static information to the perception of OCRA.

It should be noted that the subjective centre of the apparent square was located on an almost oval orbit. However, the square with an orientation of 30° and 60° induced less positional shift of the centre position. Although the current study does not enable decisive conclusions regarding this point, one possible explanation is that these orientation conditions could represent a unique case of interpolation of a global shape. Under these conditions, there were three invisible vertices (Figure 2). If participants could perceive a square or a rectangle for these stimuli, these vertices would be completed. In particular, the upper left contour in the 30° condition had no vertex defined by the background lines (only the lower right vertex was defined by the background pattern). Interestingly, the visible parts of the background at the upper left side were angled at almost 90°. If the contour was modally interpolated and located between these angles (i.e., drawn along the left oblique side of background pattern), the completed shape should be reduced in size. This might induce less positional shift (toward the veridical centre) of the centre of the shape.

### Effect of Motion Information on OCRA

In Experiment 2, the results revealed that OCRA could be cancelled out by adding point-symmetrical motion. The length of the radii of the orbit of additional rotation needed for cancellation depended on the speed of the baseline motion, such that a faster speed of rotation required a longer radius. This finding suggests that faster speed of rotation induced a greater amount of positional shift of the central rotation axis of the apparent square. Previous studies on perceptual positional shift also reported that faster motion induced more positional shift (De Valois & De Valois, 1991; Nakajima & Sakaguchi, 2016; Roulston, Self, & Zeki, 2006). OCRA would follow such characteristics of the perceptual positional shift using motion information. Note that the positional shift in OCRA was observed in the central rotation axis of the rotating shape. The effect of motion speed on OCRA, therefore, would be expected to be maintained during motion presentation (De Valois & De Valois, 1991; Ramachandran & Anstis, 1990), not instantaneously changed (Eagleman & Sejnowski, 2007; Nakajima & Sakaguchi, 2016). In this sense, the effect of motion information on OCRA might represent a kind of motion momentum (e.g., a centrifugal force).

In any case, the current results clearly demonstrate that OCRA can be induced by motion information as well as static information (interpolation of the shape). In addition, our findings revealed that the illusion consists of a fundamental positional shift induced by interpolation of the shape, and the additive positional shift induced by the rotational motion of the shape.

### “Object Motion” in OCRA

The question of what constitutes “motion information” in OCRA stimuli should be considered, because invisible parts of the square without a frame would not be expected to have a physical motion signal. One possible interpretation is that OCRA is induced by object motion (Cohen, Jain, & Zaidi, 2010), suggesting that motion information is derived from shape information (i.e., “motion from shape”). Such motion information could be based on continuous snapshots of an interpolated shape.

This continuous-snapshots hypothesis may be supported by the results of Experiment 1, which demonstrated that participants were able to judge the centre position of the apparent square. This finding suggests that the invisible and visible parts of the stimuli become integrated, resulting in the global shape. In addition, the perceived shapes did not appear to violate the principle of generic image sampling for 2D images (cf. Nakayama & Shimojo, 1992), as participants perceived the shape as a square or rectangle with a moderate size, rather than an extraordinary large or small shape. If this was not the case, the centre position within the central region of the whole stimulus would not be possible to determine (i.e., within the grey region on the background pattern). When the shape was completed as a rectangle, the central position of the apparent rectangle was located outside of that of the apparent square. This could explain why the illusory path of central rotation axis followed an oval path. Perception of a generic image might also be induced by the continuous observation of the modal/amodal shape (i.e., the temporal priming effect for perceptual completion; Yun, Hazenberg, & van Lier, 2018). In the stimuli used in the current study, the lower part of the apparent shape was frequently visible as two vertices, which would be regarded as a prime stimulus and a cue for the apparent global shape.

The results of Experiment 2 also suggested that OCRA could be induced by motion information derived from the global shape. In Experiment 2, observers judged whether the “central rotation axis” oscillated or not. The central rotational axis could not be defined by the local visible part of the stimuli, and it was only when the global shape with all vertices was completed that the central rotational axis could be judged. This suggests that the occurrence of OCRA could not be explained by the effect of local information. A previous study (Cohen et al., 2010) suggested that object motion from a shape contributed to the perception of an elastic, nonrigid pattern. The current findings appear to demonstrate the contribution of object motion to the perception of a rigid shape.

### Effect of Background Pattern on OCRA

In the current study, we used one type of stimulus that was optimized to induce OCRA, based on the authors’ observations. It is possible that the background fan-like pattern is critical for OCRA. One might argue that the fan-like (radial) pattern induces rotational motion illusion, for example, Enigma illusion in which apparent rotation or spin of something glittering can be perceived within the homogeneous-luminance ring on radial stripes (Gori, Hamburger, & Spillmann, 2006; Hamburger, 2007; Leviant, 1996; Troncoso, Macknik, Otero-Millan, & Martinez-Conde, 2008). The strength of illusory spin depends on the orientation between the homogeneous-luminance region and background pattern (Gori et al., 2006; Hamburger, 2007); T-junctions (90° orientation) can induce the illusion strongly. In the case of OCRA, however, the background pattern itself would have little effect on the induction of OCRA (Movie 2). The background pattern in Movie 2 contained filled triangles, with parameters that were identical to the original stimulus. As can be seen in Movie 2, OCRA occurs even when the background is a filled pattern, although some viewers might perceive less oscillation of OCRA in this condition. Although such a difference of background patterns caused illusory oscillation of OCRA to some degree, both stimulus configurations are sufficient for inducing OCRA. This finding suggests that the fan-like background itself is not a fundamental factor in the occurrence of OCRA. In addition, other spatial parameters (orientation of stimuli, and luminance contrast) also had little effect on OCRA induction (Movies 3–8).

**Movie 2.** An OCRA stimulus with a filled triangle of the background pattern.

**Movie 3.** An OCRA stimulus with a background pattern rotated in 90° clockwise.

**Movie 4.** An OCRA stimulus with a background pattern rotated in 90° anticlockwise.

**Movie 5.** A darker stimulus with 30% luminance of the original OCRA.

**Movie 6.** A darker stimulus with 60% luminance of the original OCRA.

**Movie 7.** A brighter stimulus with 140% luminance of the original OCRA.

**Movie 8.** A brighter stimulus with 180% luminance of the original OCRA.

As described earlier, the most critical factor in the occurrence of OCRA is whether the upper parts of stimuli are invisible or not and thus require perceptual spatial completion, resulting in an apparent square. In contrast, motion information is less effective for inducing OCRA. First, to demonstrate the effect of these factors, we presented other stimuli with a different background (Movies 9 and 10). Movie 9 shows the stimulus on a background with a longer base, and Movie 10 shows a shorter base version compared with the original stimuli. Interestingly, both stimuli induced a weaker OCRA effect. This finding suggests the existence of constraints on the visibility of the upper part of the shape. For motion information, differences in luminance contrast appear to have little effect on OCRA (Movies 5–8). The luminance contrast of stimuli is generally important for the perception of motion illusion (e.g., the stepping feet illusion; Anstis, 2003). This would support an object motion hypothesis for OCRA. Further experiments are required to investigate the effect of the invisible part of a shape. Such a study could reveal the mechanisms underlying OCRA and elucidate why the illusory orbit is oval-shaped.

**Movie 9.** An OCRA stimulus with a longer base of the background pattern.

**Movie 10.** An OCRA stimulus with a shorter base of the background pattern.

## Conclusion

In the current study, we reported the novel OCRA visual illusion and investigated its underlying mechanisms. The results revealed two main findings: (a) the centre position of a static square without a frame was shifted, and this effect depended on the orientation of the square; and (b) faster rotation induced a stronger OCRA effect. These results suggest that OCRA can be induced by both a shift of the centre position of the completed shape, and the information derived from the object motion of the global shape.
